# Work participation disparities among LGBTQ+ Australians: Insights from a nationally representative cohort study

**DOI:** 10.1371/journal.pone.0339160

**Published:** 2026-01-14

**Authors:** Dunya Tomic, Tessa Keegel, Monica O’Dwyer, Karen Walker-Bone

**Affiliations:** Monash Centre for Occupational and Environmental Health, School of Public Health and Preventive Medicine, Monash University, Melbourne, Victoria, Australia; Health Sciences, Arnavutkoy State Hospital, TÜRKIYE

## Abstract

This study examined work participation disparities among lesbian, gay, bisexual, transgender, queer, and other sexually and/or gender diverse (LGBTQ+) adults using nationally representative data from the Household, Income and Labour Dynamics in Australia (HILDA) Survey. Sexual identity data were collected in wave 20 (2020) from 14,302 participants and gender identity data in wave 23 (2023) from 13,981 participants. Multivariable regression models examined associations between sexual or gender identity and work participation measures. Sexual identity was analysed cross-sectionally (wave 20) and longitudinally (waves 20–23), while gender identity was analysed cross-sectionally (wave 23). Compared to heterosexual participants, those identifying as gay or lesbian were more likely to be unemployed (prevalence ratio [PR] 2.05, 95% CI 1.01–4.14) and less likely to work in trades or manual occupations (PR 0.55, 95% CI 0.36–0.85) or in manufacturing and construction (PR 0.40, 95% CI 0.23–0.72). Bisexual participants had higher prevalence of labour force non-participation (PR 1.91, 95% CI 1.49–2.47) and unemployment (PR 2.05, 95% CI 1.24–3.38), and were less likely to work in agriculture, forestry or mining (PR 0.24, 95% CI 0.08–0.79). Participants of other sexual identities also had higher unemployment (PR 2.78, 95% CI 1.41–5.45). Longitudinally, bisexual participants were more likely to transition out of employment (incidence rate ratio [IRR] 2.08, 95% CI 1.35–3.21) and initiate paid sick leave (IRR 1.42, 95% CI 1.17–1.71), while gay or lesbian participants were more likely to commence working from home (IRR 1.72, 95% CI 1.21–2.44). Transgender and gender diverse participants were less likely to work in manufacturing and construction (PR 0.35, 95% CI 0.17–0.75) and worked fewer hours (PR 0.88, 95% CI 0.79–0.97) than cisgender peers. These findings highlight inequalities in work participation among LGBTQ+ adults, underscoring the need for dedicated research and inclusive workplace policies.

## Introduction

Lesbian, gay, bisexual, transgender, queer, and other sexually and/or gender diverse people, collectively referred to under the umbrella term LGBTQ + , face significant disparities across various life domains, including education, healthcare access, housing, financial security, and experiences with the legal system [1–3]. The LGBTQ+ community is heterogeneous, with certain groups, such as transgender people, often facing distinct and more intense forms of discrimination and exclusion [4]. Inequities faced by LGBTQ+ individuals are shaped by both interpersonal discrimination and broader structural exclusion, often beginning early in life and accumulating over time [5]. Such disadvantages may intersect and compound across the life course, creating persistent barriers to social and economic participation [6]. A growing body of international research highlights that LGBTQ+ people are at higher risk of mental health conditions, including depression and anxiety, and are more likely to experience violence, social rejection, and minority stress [7–9]. However, much of the LGBTQ+ health research, particularly in relation to mental health and social wellbeing, has focused on children and adolescents, with comparatively less attention given to working-age adults. Emerging evidence suggests that LGBTQ+ adults face elevated risks of physical health problems, poverty, and reduced access to preventive care [10,11]. These disparities remain understudied, particularly in relation to employment and long-term socioeconomic wellbeing.

Work is a key social determinant of health, offering not only financial resources but also access to social networks, daily structure, identity, and a sense of purpose [12]. Conversely, barriers to meaningful and secure employment, such as job discrimination, exclusion from leadership roles, or limited access to entitlements, can undermine both physical and mental health [13]. Reviews of the occupational health literature have identified multiple risks experienced by LGBTQ+ workers, including poorer mental health outcomes, elevated exposure to psychosocial hazards, lack of recognition in workplace policies, and limited access to inclusive organisational cultures [14–16]. However, most existing studies group LGBTQ+ workers together rather than disaggregating by sexual or gender identity, making it difficult to draw firm conclusions about the experiences of specific subgroups. Inequities related to work for LGBTQ+ adults may contribute to job dissatisfaction, employment instability [17], and heightened stress, with potential consequences for long-term career trajectories and economic wellbeing [18]. Few studies have investigated how the workplace experiences of LGBTQ+ individuals are reflected in measurable aspects of work participation – for example, in employment status, working hours, job type, or access to sick leave [19–21]. As is the case in most countries, in Australia, national-level population-based data on these outcomes are particularly limited [22,23]. Therefore, we sought to explore the work participation of LGBTQ+ Australians using data from a nationally representative survey.

## Materials and methods

We conducted a cross-sectional and longitudinal analysis of data from the Household, Income and Labour Dynamics in Australia (HILDA) Survey to characterise work participation among a representative sample of LGBTQ+ Australians.

### Data sources

Data were sourced from the 2024 Restricted Release of the HILDA Survey, a government-funded, nationally representative panel survey of over 17,000 Australians conducted annually since 2001 [24]. Each round of data collection in the survey is referred to as a “wave”, with one wave conducted per year. HILDA uses a complex probabilistic sampling method and applies survey weights to ensure representativeness of Australians aged 15 years and older. Data collection includes a Person Questionnaire interviewer-administered through a personal interview, followed by a Self-Completion Questionnaire (SCQ) completed privately by participants and returned to the HILDA study.

### Study design

Sexual identity has been collected in the HILDA Survey every four waves since wave 12 (2012), with the most recent data available from wave 20 (2020). We first conducted a cross-sectional analysis of work participation outcomes by sexual identity using wave 20 data. To examine changes over time, we followed participants who responded to questions about their sexual identity in wave 20 and remained in the survey at wave 23 (2023). Assuming their sexual identity remained unchanged over this period, we conducted a longitudinal analysis of transitions in work outcomes.

**Fig 1:**
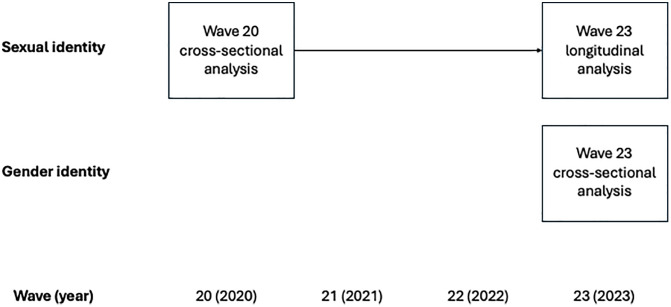
Study design flow chart.

Gender identity questions were introduced more recently in wave 22 (2022) and repeated in wave 23 (2023). Therefore, we examined work participation outcomes separately for sexually diverse and gender diverse participants. Given the limited follow-up period, we restricted analyses involving gender identity to a cross-sectional design using wave 23 data. Access to gender identity variables required approval for the HILDA 2024 Restricted Release, which includes sensitive demographic information. Our analysis was restricted to adults aged 18 years and above in wave 20 for the sexual identity analysis, and to adults aged 18 years and above in wave 23 for the gender identity analysis. An overview of the study design is presented in Fig 1.

### Sexual and gender identity measures

Sexual identity was assigned based on participants’ SCQ responses in wave 20 to the following question: “Which of the following categories best describes how you think of yourself?”. Available options included 1) heterosexual or straight; 2) gay or lesbian; 3) bisexual; 4) other; 5) unsure/don’t know; and 6) prefer not to say. Those who responded with the fifth or sixth options were excluded from the study. Regarding gender identity, transgender and gender diverse (TGD) individuals were identified through responses to two questions in wave 23, in line with recommendations from the Australian Bureau of Statistics (ABS), Australia’s national statistics agency [25]. The first question was: “What was your sex recorded at birth?”, with available options including 1) male; 2) female; and 3) other. The second question was: “How do you describe your gender?”, with response options of 1) man or male; 2) woman or female; 3) non-binary; and 4) I prefer to use a different term (please specify). From these questions, the following gender identity options were derived: 1) cisgender; 2) transgender and gender diverse; and 3) inadequately described. Those whose gender identity was inadequately described were excluded from the study.

### Work participation measures

The work participation outcomes assessed in the two cross-sectional analyses included labour force status, occupation, industry, employer type, leave, work schedule, work-from-home arrangements, supervisory responsibilities, and job satisfaction. In the HILDA Survey, labour force status was classified at both broad levels (employed, unemployed, not in the labour force) and more detailed levels (e.g., full-time and part-time employment). Occupation was coded using the International Standard Classification of Occupations third edition (ISCO-88) at the two-digit level for a participant’s main job. For this study, occupations were grouped into three categories: managers and professionals (codes 10–39), clerical and service workers (codes 40–59), and trades and manual workers (codes 60–93). Industry was classified according to the Australian and New Zealand Industrial Classification (ANZSIC) 2006 divisions and grouped into primary (agriculture, forestry, fishing, mining), secondary (manufacturing, electricity, gas, water and waste services, construction), and tertiary (all other service-related industries). Employer type was dichotomised as public sector (e.g., government business enterprises and other government employers) versus private sector (including for-profit and not-for-profit organisations). Leave was assessed based on whether participants had taken any paid sick leave or any unpaid leave in the past 12 months, as reported in the survey. Work schedule was categorised as Monday to Friday or non-standard (e.g., rotating rosters, compressed weeks, or variable workdays). Weekly work hours were analysed as a continuous variable. Work-from-home arrangements and supervisory responsibilities were recorded as binary variables. Job satisfaction was measured on a 10-point scale; due to the skewed distribution of scores, responses were dichotomised as low-to-moderate satisfaction (0–6) and high satisfaction [7–10].

The longitudinal analysis focused on work-related outcomes likely to change meaningfully over time, including labour force transitions, leave-taking behaviour, work schedule, work-from-home arrangements, and job satisfaction. More stable characteristics such as occupation and industry were excluded from our longitudinal models due to limited transitions and reduced power to detect change over the study period. To improve statistical power and capture labour market exits, we created a binary outcome variable for transition out of employment, defined as a shift from employment in wave 20 to either unemployment or not in the labour force in wave 23. Participants who were employed or actively participating in the labour force in 2020 and reported being unemployed or out of the labour force in 2023 were classified as having exited employment.

### Statistical analysis

Demographic characteristics were compared according to sexual identity and gender identity. For the sexual identity analysis, each sexual identity group (gay or lesbian, bisexual, other) was compared to those identifying as heterosexual. Gender identity was categorised as TGD versus cisgender. Group differences for categorical sociodemographic variables, including sex, educational attainment, geographic remoteness, and socioeconomic status measured by the Socio-Economic Indexes for Areas (SEIFA), were compared using survey-weighted Pearson chi-square tests (Rao-Scott adjusted). Continuous variables such as age were compared using survey-weighted linear regression. Both methods accounted for the complex survey design and sampling weights. Cross-sectional associations between sexual and gender identity and work participation outcomes were examined using Poisson regression with robust standard errors to estimate prevalence ratios (PR) with 95% confidence intervals (CI). Longitudinal associations were assessed using Poisson regression models with robust standard errors to estimate incidence rate ratios (IRR) with 95% CI. All models accounted for survey weights and stratification. Both unadjusted and adjusted models were fitted, with adjustments made for potential confounders such as age, sex, highest education level, remoteness, and SEIFA, based on sociodemographic differences between groups. We did not adjust for gender identity in the sexual identity analysis, as gender identity may lie on the causal pathway between sexual identity and work outcomes, and adjusting for it could obscure meaningful disparities. Conversely, we did not adjust for sexual identity in the gender identity analysis, as sexual identity was not collected in the same survey wave. All analyses were conducted in Stata 18.0.

### Ethical considerations and data access

This paper uses unit record data from the HILDA Survey conducted by the Australian Government Department of Social Services (DSS). The findings and views reported in this paper, however, are those of the authors and should not be attributed to the Australian Government, DSS, or any of DSS’ contractors or partners. DOI: 10.26193/J4NSZO. The data are owned by DSS and made available to researchers through the Australian Data Archive (ADA) under strict confidentiality conditions. Requirement for ethics approval specific to this study was waived by the Monash University Human Research Ethics Committee (Project ID 48041), as the HILDA Survey operates under existing ethics approval and the data are de-identified.

The primary investigator (DT) accessed the HILDA 2024 Restricted Release dataset on 23 May 2025 via the ADA under a signed Deed of Confidentiality. Due to legal and ethical restrictions imposed by the DSS and ADA, the unit record data cannot be shared publicly. Researchers may apply for access to the same dataset by contacting the Australian Data Archive (ada@anu.edu.au) or visiting https://dataverse.ada.edu.au/dataverse/hilda.

## Results

### Work participation disparities according to sexual identity

In wave 20 of the HILDA Survey (2020), a total of 14,302 participants responded to the question about their sexual identity. After applying survey weights to reflect national estimates, 94.1% (95% CI 93.5–94.6) identified as heterosexual, 1.8% (95% CI 1.6–2.2) as gay or lesbian, 2.8% (95% CI 2.4–3.2) as bisexual, and 1.3% (95% CI 1.1–1.6) as another sexual identity. Compared to heterosexual participants, those identifying as gay or lesbian were younger, had higher education levels, and were more likely to be male, reside in major cities, and reside in areas of lower socioeconomic disadvantage (Table 1). Those identifying as bisexual were more likely to be younger and female compared to heterosexual participants, while those identifying with other sexual identities were more likely to reside in areas of higher socioeconomic disadvantage.

**Table 1:** Demographic characteristics of HILDA Survey participants in wave 20 (2020), by sexual identity.

	Gay or lesbian (n = 261)	Bisexual (n = 423)	Other (n = 170)	Heterosexual (n = 13,448)
Age group (years), n (%) 18-44 45-64 ≥ 65	152 (58.2) 90 (34.5) 19 (7.3) [**p<0.001**]	355 (83.9) 52 (12.3) 16 (3.8) [**p<0.001**]	106 (62.4) 34 (20.0) 30 (17.7) [p=0.206]	6096 (45.3) 4374 (32.5) 2978 (22.1)
Mean age (years) (95% CI)	39.8 (37.8–41.8) [**p < 0.001**]	33.0 (31.2–34.7) [**p < 0.001**]	44.2 (40.6–47.9) [p = 0.066]	47.6 (47.2–48.0)
Sex*, n (%) Female Male	115 (44.1) 146 (55.9) [**p < 0.001**]	309 (73.1) 114 (27.0) [**p < 0.001**]	112 (65.9) 58 (34.1) [p = 0.101]	7166 (53.3) 6282 (46.7)
Highest education, n (%) Less than high school High school or above	20 (7.7) 241 (92.3) [**p < 0.001**]	65 (15.4) 358 (84.6) [p = 0.052]	37 (21.9) 132 (78.1) [p = 0.565]	2560 (19.0) 10,883 (81.0)
Remoteness, n (%) Major cities Regional or remote	202 (77.7) 58 (22.3) [**p = 0.011**]	294 (69.7) 128 (30.3) [p = 0.213]	120 (70.6) 50 (29.4) [p = 0.473]	9047 (67.3) 4392 (32.7)
SEIFA quintile, n (%) Quintiles 1–3 (higher disadvantage) Quintiles 4–5 (lower disadvantage)	104 (40.0) 156 (60.0) [**p < 0.001**]	273 (64.7) 149 (35.3) [p = 0.059]	111 (65.3) 59 (34.7) [**p = 0.006**]	7874 (58.6) 5565 (41.4)

*Binary variable completed by one household member on behalf of all household members; response may reflect either sex assigned at birth or current sex, depending on interpretation. CI = confidence intervals; SEIFA = socio-economic indexes for areas. Means are survey-weighted to reflect the population structure. P-values are compared to heterosexual participants and derived from survey-weighted linear regression models. *P* < 0.05 shown in bold to indicate statistical significance.

After adjusting for age, sex, highest education level, remoteness, and SEIFA, gay or lesbian participants were more likely to be unemployed (PR 2.05, 95% CI 1.01–4.14) and to work from home (PR 1.30, 95% CI 1.07–1.57) compared to heterosexual participants (Table 2). They were less likely to work in trades or manual occupations (PR 0.55, 95% CI 0.36–0.85) and to work in the secondary (manufacturing, electricity, gas, water and waste services, construction) sector (PR 0.40, 95% CI 0.23–0.72). Numerous differences in work participation outcomes were also identified between bisexual and heterosexual participants, after adjusting for age and sex: those identifying as bisexual had higher prevalence of labour force non-participation (PR 1.91, 95% CI 1.49–2.47), unemployment (PR 2.05, 95% CI 1.24–3.38), part-time employment (PR 1.22, 95% CI 1.03–1.45), and unpaid leave (PR 1.33, 95% CI 1.01–1.75). They were far less likely to work in the primary (agriculture, forestry, fishing, mining) sector (PR 0.24, 95% CI 0.08–0.79). After adjustment for SEIFA, those with other sexual identities were more likely to be unemployed (PR 2.78, 95% CI 1.41–5.45), part-time employed (PR 1.57, 95% CI 1.16–2.12), work in the public sector (PR 1.15, 95% CI 1.06–1.24), and work non-standard schedules (PR 1.60, 95% CI 1.36–1.88) compared to those identifying as heterosexual.

**Table 2:** Prevalence ratios for work participation outcomes by sexual identity: cross-sectional analysis of HILDA wave 20 (2020) data.

Variable	Outcome	PR (gay/lesbian vs. heterosexual)^a^	PR (bisexual vs. heterosexual)^b^	PR (other vs. heterosexual)^c^
Labour force status	Not in labour force (ref: in labour force)	**Unadj: 0.53 (0.39–0.71)** Adj: 0.89 (0.67–1.17)	Unadj: 1.04 (0.83–1.30) **Adj: 1.91 (1.49–2.47)**	Unadj: 1.09 (0.84–1.41) Adj: 1.04 (0.80–1.35)
	Unemployed (ref: employed)	**Unadj: 2.15 (1.05–4.38)** **Adj: 2.05 (1.01–4.14)**	**Unadj: 2.55 (1.54–4.26)** **Adj: 2.05 (1.24–3.38)**	**Unadj: 2.84 (1.48–5.45)** **Adj: 2.78 (1.41–5.45)**
	Part-time employed (ref: full-time employed)	Unadj: 0.83 (0.60–1.15) Adj: 0.95 (0.69–1.33)	**Unadj: 1.38 (1.15–1.66)** **Adj: 1.22 (1.03–1.45)**	**Unadj: 1.57 (1.16–2.12)** **Adj: 1.57 (1.16–2.12)**
Occupation	Clerical/service work (ref: manager/professional)	Unadj: 0.81 (0.59–1.10) Adj: 0.97 (0.73–1.31)	Unadj: 1.23 (0.99–1.54) Adj: 1.02 (0.83–1.26)	Unadj: 1.35 (0.92–1.99) Adj: 1.22 (0.81–1.84)
	Trades/manual work (ref: manager/professional)	**Unadj: 0.53 (0.34–0.83)** **Adj: 0.55 (0.36–0.85)**	**Unadj: 0.61 (0.41–0.89)** Adj: 0.76 (0.49–1.16)	Unadj: 1.57 (1.00–2.46) Adj: 1.35 (0.86–2.11)
Industry	Primary sector (ref: tertiary)	Unadj: 0.54 (0.15–1.91) Adj: 0.63 (0.19–2.09)	**Unadj: 0.20 (0.06–0.62)** **Adj: 0.24 (0.08–0.79)**	Unadj: 0.74 (0.27–2.04) Adj: 0.67 (0.24–1.88)
	Secondary sector (ref: tertiary)	**Unadj: 0.46 (0.26–0.81)** **Adj: 0.40 (0.23–0.72)**	Unadj: 0.57 (0.31–1.05) Adj: 0.69 (0.38–1.26)	Unadj: 1.01 (0.59–1.72) Adj: 0.97 (0.56–1.66)
Employer type	Private sector work (ref: public sector)	Unadj: 1.01 (0.93–1.09) Adj: 0.98 (0.91–1.06)	Unadj: 1.01 (0.92–1.09) Adj: 1.00 (0.93–1.08)	**Unadj: 1.15 (1.06–1.25)** **Adj: 1.15 (1.06–1.24)**
Leave	Any paid sick leave (ref: no paid sick leave)	Unadj: 1.12 (0.95–1.33) Adj: 1.14 (0.96–1.35)	Unadj: 0.97 (0.83–1.14) Adj: 0.93 (0.79–1.09)	Unadj: 0.83 (0.60–1.16) Adj: 0.84 (0.60–1.16)
	Any unpaid leave (ref: no unpaid leave)	Unadj: 0.94 (0.67–1.32) Adj: 0.98 (0.70–1.37)	**Unadj: 1.60 (1.21–2.09)** **Adj: 1.33 (1.01–1.75)**	Unadj: 1.28 (0.84–1.97) Adj: 1.25 (0.81–1.92)
Work schedule	Non-standard work schedule (ref: Mon-Fri)	Unadj: 0.96 (0.76–1.21) Adj: 1.04 (0.82–1.31)	Unadj: 1.16 (0.98–1.38) Adj: 1.13 (0.96–1.33)	**Unadj: 1.65 (1.40–1.95)** **Adj: 1.60 (1.36–1.88)**
	Hours per week	Unadj: 1.02 (0.96–1.08) Adj: 0.99 (0.94–1.05)	**Unadj: 0.91 (0.85–0.97)** Adj: 0.94 (0.89–1.00)	Unadj: 0.90 (0.81–1.00) Adj: 0.90 (0.81–1.00)
Work from home	Work from home (ref: no work from home)	**Unadj: 1.40 (1.16–1.69)** **Adj: 1.30 (1.07–1.57)**	Unadj: 0.92 (0.71–1.19) Adj: 1.02 (0.78–1.32)	Unadj: 0.77 (0.50–1.17) Adj: 0.86 (0.57–1.29)
Supervision	Supervisor role (ref: no supervisor role)	Unadj: 0.87 (0.69–1.09) Adj: 0.82 (0.65–1.03)	Unadj: 0.78 (0.62–0.97) Adj: 0.83 (0.66–1.04)	Unadj: 1.00 (0.70–1.43) Adj: 1.04 (0.73–1.49)
Job satisfaction	Low-moderate job satisfaction (ref: high)	Unadj: 1.11 (0.64–1.93) Adj: 1.12 (0.65–1.93)	**Unadj: 1.43 (1.03–2.00)** Adj: 1.34 (0.95–1.89)	Unadj: 1.03 (0.58–1.82) Adj: 1.02 (0.57–1.80)

Adj = adjusted models; CI = confidence intervals; PR = prevalence ratio; Unadj = unadjusted models. ^a^Adjusted for age, sex, highest education level, remoteness, and SEIFA. ^b^Adjusted for age and sex. ^c^Adjusted for SEIFA. All models were estimated using Poisson regression with robust standard errors, accounting for survey weights and the stratified sampling design. PRs with 95% confidence intervals not crossing 1 are shown in bold to indicate statistical significance.

For the longitudinal analysis, 12,264 of those who had reported a sexual identity in wave 20 remained in the HILDA Survey at wave 23 (2023). Weighted estimates showed a very similar distribution: 94.4% (95% CI 93.8–94.9) heterosexual, 1.7% (95% CI 1.5–2.0) gay or lesbian, 2.6% (95% CI 2.3–3.0) bisexual, and 1.3% (95% CI 1.0–1.6) other. As in the cross-sectional analysis, gay or lesbian participants were younger, had higher education levels, and were more likely to be male, reside in major cities, and reside in areas of lower socioeconomic disadvantage (S1 Table). Those identifying as bisexual were on average younger and more likely to be female compared to their heterosexual counterparts, while those of other sexual identity more commonly resided in areas of higher socioeconomic disadvantage. After adjusting for age, sex, highest education level, remoteness, and SEIFA, gay or lesbian participants were more likely to commence working from home during follow-up (IRR 1.72, 95% CI 1.21–2.44) compared to heterosexual participants (Table 3). Those identifying as bisexual made more frequent transitions out of employment (IRR 2.08, 95% CI 1.35–3.21) and made new use of paid sick leave (IRR 1.42, 95% CI 1.17–1.71) compared to those identifying as heterosexual, after adjusting for age and sex. No differences in work participation outcomes between those of other sexual identities and heterosexual participants were identified in the longitudinal analysis.

**Table 3:** Incidence rate ratios for work participation outcomes by sexual identity: longitudinal analysis of HILDA wave 20 (2020) to wave 23 (2023) data.

Variable	Outcome	IRR (gay/lesbian vs. heterosexual)^a^	IRR (bisexual vs. heterosexual)^b^	IRR (other vs. heterosexual)^c^
Labour force status	Transition to not employed (ref: remained employed)	Unadj: 0.80 (0.48–1.34) Adj: 0.98 (0.59–1.62)	Unadj: 1.50 (0.96–2.36) **Adj: 2.08 (1.35–3.21)**	Unadj: 1.31 (0.58–2.93) Adj: 1.25 (0.55–2.80)
	Transition to part-time work (ref: remained full-time)	Unadj: 0.88 (0.44–1.78) Adj: 1.07 (0.55–2.08)	Unadj: 1.32 (0.70–2.47) Adj: 1.19 (0.66–2.14)	Unadj: 0.73 (0.22–2.39) Adj: 0.73 (0.22–2.38)
Leave	New use of paid sick leave (ref: no use at either timepoint)	Unadj: 1.25 (0.80–1.93) Adj: 1.15 (0.76–1.75)	**Unadj: 1.84 (1.55–2.18)** **Adj: 1.42 (1.17–1.71)**	Unadj: 0.86 (0.45–1.63) Adj: 0.86 (0.45–1.62)
	New use of unpaid leave (ref: no use at either timepoint)	Unadj: 1.19 (0.69–2.03) Adj: 1.22 (0.72–2.06)	Unadj: 1.40 (0.97–2.03) Adj: 1.13 (0.79–1.61)	Unadj: 1.34 (0.64–2.81) Adj: 1.31 (0.62–2.77)
Work schedule	Onset of non-standard schedule (ref: remained Mon-Fri)	Unadj: 0.85 (0.50–1.44) Adj: 0.99 (0.59–1.68)	Unadj: 1.07 (0.59–1.96) Adj: 0.99 (0.55–1.78)	N/A^d^
	Onset of reduced working hours (<35 hours/week)	Unadj: 0.83 (0.49–1.41) Adj: 0.99 (0.59–1.66)	Unadj: 1.42 (0.92–2.20) Adj: 1.46 (0.95–2.23)	Unadj: 0.77 (0.31–1.89) Adj: 0.76 (0.31–1.85)
Work from home	Onset of working from home (ref: remained not working from home)	**Unadj: 1.87 (1.30–2.70)** **Adj: 1.72 (1.21–2.44)**	Unadj: 1.40 (0.91–2.16) Adj: 1.29 (0.84–1.99)	Unadj: 0.55 (0.21–1.43) Adj: 0.60 (0.24–1.49)
Supervision	Transition to supervisor role (ref: remained non-supervisory)	Unadj: 1.25 (0.78–2.02) Adj: 1.21 (0.77–1.91)	Unadj: 1.26 (0.90–1.78) Adj: 1.09 (0.77–1.56)	Unadj: 0.36 (0.13–1.00) Adj: 0.36 (0.13–1.00)
Job satisfaction	Onset of low-moderate job satisfaction (ref: remained high)	**Unadj: 1.73 (1.04–2.86)** Adj: 1.68 (1.00–2.82)	Unadj: 1.70 (1.07–2.70) Adj: 1.54 (0.97–2.45)	Unadj: 1.10 (0.45–2.68) Adj: 1.08 (0.44–2.65)

Adj = adjusted models; CI = confidence intervals; IRR = incidence rate ratio; Unadj = unadjusted models. ^a^Adjusted for age, sex, highest education level, remoteness, and SEIFA. ^b^Adjusted for age and sex. ^c^Adjusted for SEIFA. ^d^Estimate not calculated due to small cell counts among respondents with other sexual identity. All models were estimated using Poisson regression with robust standard errors, accounting for survey weights and the stratified sampling design. Outcomes represent transitions (onset or change) between waves 20 and 23. IRRs with 95% confidence intervals not crossing 1 are shown in bold to indicate statistical significance.

### Work participation disparities according to gender identity

In wave 23 of the HILDA Survey (2023), 13,981 participants responded to questions about their gender identity. After applying survey weights to reflect national estimates, 98.8% (95% CI 98.5–99.0) identified as cisgender and 1.2% (95% CI 1.0–1.5) as TGD. Compared to those identifying as cisgender, TGD participants were younger on average (Table 4). After adjusting for age, TGD participants were less likely to work in the secondary (manufacturing, electricity, gas, water and waste services, construction) sector (PR 0.35, 95% CI 0.17–0.75) and reported working fewer hours per week (PR 0.88, 95% 0.79–0.97) (Table 5). No other differences in work participation outcomes by gender identity were observed.

**Table 4:** Demographic characteristics of HILDA Survey participants in wave 23 (2023), by gender identity.

	Transgender and gender diverse (n = 163)	Cisgender (n = 13,818)	P-value
Age group (years), n (%) 18-44 45-64 ≥ 65	126 (77.3) 25 (15.2) 12 (7.4)	6253 (45.3) 4251 (30.8) 3314 (24.0)	**<0.001**
Mean age (years) (95% CI)	35.2 (32.6–37.7)	47.4 (47.0–47.9)	**<0.001**
Highest education, n (%) Less than high school High school or above	25 (15.3) 138 (84.7)	2584 (18.7) 11,227 (81.3)	0.789
Remoteness, n (%) Major cities Inner regional Outer/remote/very remote	113 (69.3) 38 (23.3) 12 (7.4)	9265 (67.2) 3123 (22.6) 1407 (10.2)	0.918
SEIFA quintile, n (%) 1 (most disadvantaged) 2 3 4 5 (least disadvantaged)	39 (23.9) 26 (16.0) 33 (20.3) 34 (20.9) 31 (19.0)	2772 (20.1) 2748 (19.9) 2787 (20.2) 2812 (20.4) 2676 (19.4)	0.954

CI = confidence intervals; SEIFA = socio-economic indexes for areas. Means are survey-weighted to reflect the population structure. P-values are from survey-weighted linear regression models. *P* < 0.05 shown in bold to indicate statistical significance.

**Table 5:** Prevalence ratios for work participation outcomes by gender identity: cross-sectional analysis of HILDA wave 23 (2023) data.

Variable	Outcome	Unadjusted PR (transgender and gender diverse vs. cisgender) (95% CI)	Age-adjusted PR (transgender and gender diverse vs. cisgender) (95% CI)
Labour force status	Not employed (ref: employed)	0.81 (0.59–1.11)	1.35 (1.00–1.84)
	Part-time employed (ref: full-time employed)	1.20 (0.85–1.71)	1.19 (0.84–1.69)
Occupation	Clerical/service work (ref: manager/professional)	1.24 (0.86–1.79)	1.11 (0.78–1.58)
	Trades/manual work (ref: manager/professional)	0.91 (0.50–1.66)	0.89 (0.49–1.63)
Industry	Secondary sector (ref: tertiary)	**0.36 (0.17–0.75)**	**0.35 (0.17–0.75)**
Employer type	Private sector work (ref: public sector)	0.84 (0.66–1.07)	0.83 (0.65–1.05)
Leave	Any paid sick leave (ref: no paid sick leave)	0.94 (0.73–1.22)	0.92 (0.71–1.19)
	Any unpaid leave (ref: no unpaid leave)	1.34 (0.89–2.02)	1.16 (0.78–1.72)
Work schedule	Non-standard work schedule (ref: Mon-Fri)	1.24 (0.94–1.65)	1.24 (0.93–1.64)
	Hours per week	**0.87 (0.78–0.97)**	**0.88 (0.79–0.97)**
Work from home	Work from home (ref: no work from home)	0.70 (0.44–1.09)	0.77 (0.49–1.22)
Supervision	Supervisor role (ref: no supervisor role)	0.80 (0.52–1.22)	0.82 (0.53–1.25)
Job satisfaction	Low-moderate job satisfaction (ref: high)	1.44 (0.81–2.57)	1.34 (0.76–2.39)

All models were estimated using Poisson regression with robust standard errors, accounting for survey weights and the stratified sampling design. PRs with 95% confidence intervals not crossing 1 are shown in bold to indicate statistical significance.

## Discussion

Using nationally representative, longitudinal data from the HILDA Survey, this study identified disparities in work participation outcomes by sexual and gender identity among Australian adults. Compared to heterosexual participants, those identifying as gay or lesbian were more likely to be unemployed and to work from home, and less likely to work in trades, manual occupations, or the secondary sector (manufacturing, construction, utilities). Bisexual participants were more likely to be outside the labour force, unemployed, and engaged in part-time or unpaid leave arrangements, and were under-represented in the primary industries of agriculture, forestry, fishing, and mining. Participants identifying with other sexual identities were more likely to be unemployed, work part-time, work in the public sector, and have non-standard work schedules. In longitudinal analyses, gay and lesbian participants were more likely than their heterosexual peers to commence working from home between 2020 and 2023, while bisexual participants had higher rates of transition out of employment and new use of paid sick leave. Differences by gender identity were less pronounced; however, this study may not have detected differences reported elsewhere [26], likely due to the small TGD sample size and limited statistical power. Additionally, underreporting of gender diversity may occur, for example among communities with low acceptance of gender diversity, which could further limit detection of disparities in survey data.

The proportion of LGBTQ+ respondents in this study broadly aligns with national population estimates. According to the ABS, in 2022, 3.6% of Australians aged 16 years and over identified as lesbian, gay, bisexual, or another non-heterosexual orientation, and 0.9% as TGD [27]. Our weighted cross-sectional estimates of 5.9% and 1.2%, respectively, are slightly higher, which may be driven by several factors. Notably, in the HILDA Survey, questions on sexual and gender identity are included in a self-administered questionnaire, which could encourage greater disclosure compared with face-to-face ABS interviews. Differences in weighting processes, years of data collection (2020 and 2023 in our study vs. 2022 in the ABS data), and the small size of TGD populations making their estimates more sensitive to sampling variability, may also in part account for these differences.

Several factors, at the personal, workplace, and societal levels, might contribute to these disparities in work participation outcomes for LGBTQ+ workers [28]. Notably, gay or lesbian participants in our study were less likely than heterosexual peers to leave school before completing high school, indicating greater persistence in pursuing secondary education. This suggests a possible strategic choice among gay or lesbian individuals to attain at least high school education in order to access occupations perceived as less stigmatised or more inclusive than diploma- or trade-based pathways, which often lead to heteronormative workplace cultures. This contrasts with some prior research suggesting poorer educational outcomes among LGBTQ+ populations [29]. This discrepancy may reflect unresolved complexities in research on LGBTQ+ educational attainment, sample-specific characteristics, or limitations in survey representativeness. It is also possible that more highly educated LGBTQ+ individuals are more willing or able to disclose their identities in large-scale surveys. Self-disclosure hesitancy remains an important limitation of survey-based research [30], as individuals who choose not to disclose may differ systematically from those who do – for instance, they may have greater concerns about privacy, fear stigma or discrimination, or feel unsafe identifying as LGBTQ+ in certain contexts. Consequently, these dynamics may contribute to underestimation of disparities within population-level datasets.

Despite higher educational attainment, gay or lesbian participants had poorer work participation outcomes in both cross-sectional and longitudinal analyses. This discrepancy suggests the presence of systemic and structural barriers beyond individual qualifications, which aligns with previous evidence showing the negative impacts of workplace discrimination, exclusion, and harassment on LGBTQ+ employees’ career trajectories [31]. At the personal level, these outcomes may relate to the mental and physical health impacts of minority stress [32]. Numerous work participation disparities were also observed for bisexual participants. The term “double discrimination” has been used to describe the prejudice against bisexuals from both heterosexuals and the LGBTQ+ community [33]. Community studies of bisexuals have shown higher rates of depression, anxiety, suicidality, and substance abuse, compared to monosexuals [7,34]. However, research on the specific workplace experiences of bisexuals is limited. For example, one small US survey found that over 50% of those identifying as bisexual reported employment discrimination at some point in their lives [35], which may in part explain the adverse work participation outcomes observed in our study. The increased likelihood of transitioning out of employment among bisexual individuals might reflect burnout or difficulties sustaining long-term career progression in unsupportive environments [36], as well as cumulative disadvantage at the societal level [37]. Such cumulative disadvantage may manifest not only as stress-related health conditions but also as career attrition, whereby exposure to repeated marginalisation prompts withdrawal from certain roles, sectors, or leadership pathways.

LGBTQ+ workers may face exclusion from industries that offer stable employment, higher wages, and clearer career pathways. Our findings show that both sexually and gender diverse participants were less likely to work in traditionally higher-paid sectors such as manufacturing, utilities, and construction. Additionally, bisexual participants were less likely to be employed in resource-based industries like agriculture, forestry, fishing, and mining – this difference for gender diverse participants may have been undetected due to smaller sample size and limited statistical power. These patterns likely reflect a combination of active exclusion, through discriminatory hiring or workplace cultures, and anticipatory avoidance, where LGBTQ+ individuals choose to steer away from male-dominated or culturally conservative sectors due to perceived or experienced risks of discrimination, microaggressions, or harassment [32]. Given the strong gendered nature of these sectors and potential limitations in the survey’s representation of gender diversity, these results should be interpreted with some caution. Industries such as manufacturing, utilities, and construction are often characterised by entrenched cisgender masculine norms, low workforce diversity, and informal recruitment networks, which can create environments where LGBTQ+ workers feel unwelcome or unsafe [38]. In response, anticipatory avoidance may lead LGBTQ+ individuals to self-select out of some industries to protect their wellbeing, even in the absence of overt discrimination. Consequently, many may gravitate towards potentially safer or more flexible roles in service-oriented sectors [31]. Despite offering greater psychological safety, these roles often provide less stability, fewer benefits, and limited advancement opportunities, thereby perpetuating structural inequalities in occupational outcomes.

Societal factors, including prevailing social attitudes and the availability of supportive policies, further shape these employment patterns. Prior research suggests that an LGBTQ + -friendly work climate is often more important for cisgender women and gender minorities within the LGBTQ+ community than for cisgender men, who tend to prioritise income more highly [36]. The need to protect psychological safety may drive preferences for an inclusive workplace over higher-paying roles with career progression opportunities. While our study’s limited TGD sample size may constrain the detection of some disparities, gender-diverse individuals are often structurally underrepresented in general population studies not designed to centre LGBTQ+ experiences, contributing to the invisibility of their employment challenges in quantitative research. Although our findings for TGD participants were limited, this should not be interpreted as evidence of reduced vulnerability; rather, it reflects ongoing data limitations that constrain the ability to meaningfully capture TGD workforce exclusion. Overall, our findings of reduced participation in specific sectors and fewer hours worked align with prior research highlighting employment vulnerabilities among gender-diverse populations [39,40]. Although our sample of those with “other” sexual identities was also relatively small, we found that they had reduced labour force participation, higher unemployment, and higher likelihood of part-time employment. These findings underline the importance of co-designing research with people who hold these identities, as existing survey frameworks may inadequately capture the diversity of sexual identities such as asexual, pansexual, queer, or questioning. Without inclusive and community-informed measures, important differences in workplace participation and experiences may remain obscured, limiting the evidence base needed to inform equitable employment policy.

Our study’s strengths include its use of a nationally representative longitudinal sample from the HILDA Survey, relatively granular sexual and gender identity data, and comprehensive work outcome metrics, all of which enhance the generalisability and robustness of our findings. Limitations of the study are that sexual and gender identity data were not collected in all waves of the HILDA Survey, with gender identity data in particular limited to a single cross-sectional data point. The small TGD sample size reduces statistical power in our analysis, increasing the likelihood of Type II error; findings for this group should therefore be interpreted cautiously. Sexual identity was assumed to remain stable between 2020 and 2023, which may not reflect the fluidity of sexual identity over time, particularly for younger cohorts. Potential social desirability bias may lead to underreporting of non-cisgender and non-heterosexual identities, both within the self-reported and interviewer-collected data, affecting the accuracy of our findings. Furthermore, the reliance on binary employment outcomes (e.g., employed vs. unemployed) may not capture more nuanced underemployment or job mismatch experiences, which are particularly relevant to LGBTQ+ individuals working in flexible, self-employed or precarious roles [41]. Similarly, dichotomising job satisfaction may have reduced variability in responses; however, this was necessary given the small number of responses at each level of job satisfaction among specific sexual identities. Finally, given the exploratory scope and number of outcomes examined, we did not adjust for multiple comparisons; as such, some findings may reflect chance associations and should be interpreted with appropriate caution.

Future research should prioritise collection of sexual and gender identity data from diverse population-based sources to enable robust longitudinal analyses. Complementary studies collecting employer-level workplace data or conducting longitudinal qualitative research could identify specific workplace practices that facilitate or hinder inclusion. Expanding our understanding of mediators of exclusion such as discrimination, poor mental health, and lack of family support is essential. Additional focus on protective factors, such as access to affirming leadership, union representation, or inclusive organisational cultures, may offer insight into strategies that mitigate disadvantage. Intersectional analyses considering ethnicity, disability, and rurality are particularly warranted to capture compounding disadvantages experienced by LGBTQ+ people from ethnically or culturally diverse communities, those with disabilities, and individuals in regional or remote areas [42–44]. Future studies would also benefit from examining income trajectories, job satisfaction across different organisational contexts, and perceived support from colleagues or managers. Further attention is also needed to explore the intersection between sexual and gender identity, as individuals who identify as both sexually and gender diverse may experience compounded forms of exclusion or discrimination in the workplace. While we analysed sexual and gender identity separately due to causal considerations and data availability, with gender identity questions only introduced in later waves, future research should investigate their combined effects to better capture the experiences of LGBTQ+ individuals living at these intersections.

The combined evidence from our study suggests an occupational stratification pattern that constrains economic security and upward mobility for LGBTQ+ workers. This is reflected in their underrepresentation in sectors associated with career stability (e.g., manufacturing, electricity, gas, water and waste services, construction). The findings from our study emphasise the need for more inclusive and supportive policies for sexually and gender diverse workers. These include proactive workplace inclusion strategies, workplace mental health supports and anti-discrimination efforts. Increased training for employers, managers and other employees on LGBTQ+ inclusion can improve retention and job satisfaction, by educating the workforce on the challenges faced by LGBTQ+ workers, potentially building a supportive and inclusive occupational environment which benefits all workers. However, workplace efforts alone may be insufficient without broader cultural change. Improving access to inclusive education pathways and career planning support is also essential to ensure that LGBTQ+ individuals can pursue and sustain meaningful employment across diverse sectors. Reducing stigma, improving representation, and advancing structural reform remain critical to ensuring equity for LGBTQ+ workers.

## Supporting information

S1 Table Demographic characteristics of participants who reported sexual identity in wave 20 (2020) and remained in the HILDA Survey at wave 23 (2023). (DOCX)
