# Supplementary material for: Work participation disparities among LGBTQ+ Australians: Insights from a nationally representative cohort study
Source: PLoS One. 2026 Jan 14;21(1):e0339160. doi: 10.1371/journal.pone.0339160 (PMC12803448; doi:10.1371/journal.pone.0339160)
Supplement: S1 Table — (DOCX) [file pone.0339160.s001.docx]

|  | **Gay or lesbian (n=222)** | **Bisexual (n=346)** | **Other (n=141)** | **Heterosexual (n=11,555)** |
| --- | --- | --- | --- | --- |
| Age group (years), n (%)  18-44  45-64  ≥65 | 111 (50.0)  84 (37.8)  27 (12.2) [**p<0.001**] | 272 (78.6)  51 (14.7)  23 (6.7) [**p<0.001**] | 83 (58.9)  29 (20.6)  29 (20.6) [p=0.141] | 4643 (40.2)  3857 (33.4)  3055 (26.4) |
| Mean age (years) (95% CI) | 43.4 (41.0 – 45.9) [**p<0.001**] | 36.4 (34.5 – 38.4) [**p<0.001**] | 47.1 (42.8 – 51.3) [p=0.151] | 50.2 (49.7 – 50.6) |
| Sex*, n (%)  Female  Male | 101 (45.5)  121 (54.5) [**p<0.001**] | 249 (72.0)  97 (28.0) [**p=0.006**] | 91 (64.5)  50 (35.5) [p=0.263] | 6174 (53.4)  5381 (46.6) |
| Highest education, n (%)  Less than high school  High school or above | 17 (7.7)  203 (92.3)  [**p=0.001**] | 51 (14.9)  292 (85.1) [p=0.324] | 27 (19.7)  110 (80.3) [p=0.848] | 2013 (17.7)  9362 (82.3) |
| Remoteness, n (%)  Major cities  Regional or remote | 173 (78.3)  48 (21.7) [**p=0.039**] | 241 (69.9)  104 (30.1) [p=0.206] | 100 (70.9)  41 (29.1) [p=0.570] | 7701 (66.8)  3832 (33.2) |
| SEIFA quintile, n (%)  Quintiles 1-3 (higher disadvantage)  Quintiles 4-5 (lower disadvantage) | 101 (45.7)  120 (54.3) [**p=0.008**] | 226 (65.5)  119 (34.5) [p=0.109] | 95 (67.4)  46 (32.6) [**p=0.010**] | 6807 (59.0)  4726 (41.0) |

*Binary variable completed by one household member on behalf of all household members; response may reflect either sex assigned at birth or current sex, depending on interpretation. CI = confidence intervals; SEIFA = socio-economic indexes for areas. Means are survey-weighted to reflect the population structure. P-values are compared to heterosexual participants and derived from survey-weighted linear regression models. P < 0.05 shown in bold to indicate statistical significance.
