# Supplementary material for: Work participation disparities among LGBTQ+ Australians: Insights from a nationally representative cohort study
Source: PLoS One. 2026 Jan 14;21(1):e0339160. doi: 10.1371/journal.pone.0339160 (PMC12803448; doi:10.1371/journal.pone.0339160)
Supplement: Attachment — Submitted filename: Reviewer responses 27.10.25.docx [file pone.0339160.s003.docx]

27 Oct 2025

Dr Ozan Karadeniz

Academic Editor

PLOS ONE

Dear Dr Karadeniz,

Thank you for providing this helpful feedback, and for the opportunity to revise our manuscript. We have made the requested changes and address each of the editorial and reviewers’ comments below.

**Associate Editor:**

1. The statistical methodology is sound and appropriate for the research questions. The main limitation is under-adjustment for confounders and limited power in the gender identity subgroup. Results should be interpreted with caution regarding causality and subgroup generalizability. Limitations / Points to Flag:

- Limited confounder adjustment – only age and education were included; other structural factors could influence results.
- Small TGD sample size – increases risk of Type II error; underpowered for some outcomes.
- Assumption of stable sexual identity over 3 years may not reflect reality.
- Job satisfaction dichotomization loses nuance; ordinal or linear models could provide more detail.
- Potential multiple testing issue – many outcomes tested, but no adjustment for multiple comparisons (risk of false positives).

RESPONSE

**Thank you for these constructive suggestions. We have revised the Limitations section to more clearly emphasise the need for cautious interpretation and to elaborate on the issues raised, including statistical power, potential Type II error, and multiple testing. In response to Reviewer 2’s comments, all sexual identity analyses were re-run with disaggregated sexual identity categories and additional covariate adjustment for age, sex, highest education level, remoteness, and area-level socioeconomic position (SEIFA), based on variables showing significant between-group differences. Adjusting only for covariates that differed meaningfully between groups helps to prevent statistical over-adjustment, which can remove variance intrinsic to sexual identity and obscure genuine disparities.**

***Page 22, line 451: “****The small TGD sample size reduces statistical power in our analysis, increasing the likelihood of Type II error; findings for this group should therefore be interpreted cautiously. Sexual identity was assumed to remain stable between 2020 and 2023, which may not reflect the fluidity of sexual identity over time, particularly for younger cohorts. Potential social desirability bias may lead to underreporting of non-cisgender and non-heterosexual identities, both within the self-reported and interviewer-collected data, affecting the accuracy of our findings. Furthermore, the reliance on binary employment outcomes (e.g., employed vs. unemployed) may not capture more nuanced underemployment or job mismatch experiences, which are particularly relevant to LGBTQ+ individuals working in flexible, self-employed or precarious roles (1). Similarly, dichotomising job satisfaction may have reduced variability in responses; however, this was necessary given the small number of responses at each level of job satisfaction among specific sexual identities. Finally, given the exploratory scope and number of outcomes examined, we did not adjust for multiple comparisons; as such, some findings may reflect chance associations and should be interpreted with appropriate caution.”*

**Reviewer 1:**

1. Page 7, Line 144: Recommend including an example or two of responses that were “inadequately described.”

RESPONSE

**In light of this suggestion, we consulted with the HILDA Survey data custodians regarding examples of responses that were “inadequately described”; they advised that, due to confidentiality requirements, it is not possible to provide specific examples. As such, we have not included examples in the manuscript.**

2. Page 9: I appreciated the explanation for why adjustments were not made for gender identity in the sexual identity analysis and similarly for sexual identity in the gender identity analysis. The rationale was clear and proactively addressed a question readers may have (i.e., what about individuals who identify as both non-heterosexual and TGD).

RESPONSE

Thank you for this positive feedback.

3. Page 10, Lines 221-224 and Page 14, 279-280: How does the study’s sample sizes (% of non-heterosexual and TGD) compare to national population estimates? If possible, this reviewer recommends including that detail.

RESPONSE

We have now included a paragraph in the Discussion comparing LGBTQ+ population estimates in our study to those reported by the Australian Bureau of Statistics, noting broad alignment and possible reasons for differences.

*Page 18, line 351: “The proportion of LGBTQ+ respondents in this study broadly aligns with national population estimates. According to the ABS, in 2022, 3.6% of Australians aged 16 years and over identified as lesbian, gay, bisexual, or another non-heterosexual orientation, and 0.9% as TGD (2). Our weighted cross-sectional estimates of 5.9% and 1.2%, respectively, are slightly higher, which may be driven by several factors. Notably, in the HILDA Survey, questions on sexual and gender identity are included in a self-administered questionnaire, which could encourage greater disclosure compared with face-to-face ABS interviews. Differences in weighting processes, years of data collection (2020 and 2023 in our study vs. 2022 in the ABS data), and the small size of TGD populations making their estimates more sensitive to sampling variability, may also in part account for these differences.”*

4. Page 17, Line 329: The authors briefly reference self-disclosure hesitancy in surveys. This reviewer recommends expanding this as a potential limitation of the study because the findings do not capture those who chose not to self-disclose. It also may be worthwhile to include commentary about reasons for hesitancy to self-disclose on surveys.

RESPONSE

We agree that self-disclosure hesitancy represents an important limitation of survey-based research. We have expanded the discussion to include this point and to outline potential reasons for nondisclosure, such as privacy concerns, fear of stigma or discrimination, and perceived lack of safety in certain contexts.

*Page 19, line 374: “Self-disclosure hesitancy remains an important limitation of survey-based research (3), as individuals who choose not to disclose may differ systematically from those who do – for instance, they may have greater concerns about privacy, fear stigma or discrimination, or feel unsafe identifying as LGBTQ+ in certain contexts. Consequently, these dynamics may contribute to underestimation of disparities within population-level datasets.”*

**Reviewer 2:**

1. In line 77, instead of “facing”, I think the authors mean “regarding”.

RESPONSE

We have updated the wording to “experienced by” to clarify that these risks are those that LGBTQ+ workers encounter, rather than risks merely discussed in the literature.

*Page 3, line 74: “Reviews of the occupational health literature have identified multiple risks experienced by LGBTQ+ workers, including poorer mental health outcomes, elevated exposure to psychosocial hazards, lack of recognition in workplace policies, and limited access to inclusive organisational cultures (4-6).”*

2. Regarding the categorization of the sexually diverse participants, I find it contradictory and rather invisibilizing that in the introduction, the authors point out the fact that previous studies, and I quote them literally: “group LGBTQ+ workers together rather than disaggregating by sexual or gender identity, making it difficult to draw firm conclusions about the experiences of specific subgroups” and when they proceed to the analysis section, they go and do the same thing. I think that if the authors are writing a manuscript titled “Work participation disparities among LGBTQ+ Australians: insights from a nationally representative cohort study”, it doesn’t make sense that 94% of the sample they use is composed of heterosexual participants. I understand that LGBTQ+ people compose about 4.5% of the population in Australia, and obviously, heterosexual people are going to be the majority. However, as a researcher who also works in the field of sexual diversity, I believe that this article is poorly conceived. I think it would make much more sense to focus on the number of “non-heterosexual” participants (which I see is 929 people), divide them so that each category (bisexuals, lesbians, gays) can have the visibility it deserves, and take an equivalent sample of heterosexual people for comparison. In this way, the article would take on a more positive connotation than the ‘otherness’ it currently appears to have. I think the authors should consider this and modify it.

RESPONSE

Thank you for raising this important point. We agree that disaggregating sexual identity subgroups is essential to avoid obscuring differences within the LGBTQ+ population. In response, we have now redone all sexual identity analyses to present results separately for each subgroup (gay or lesbian, bisexual, and other sexual identities) compared with heterosexual participants. We have updated the relevant tables (Tables 1-3; S1 Table) and text accordingly. We believe this approach is preferable to creating a matched heterosexual sample, as it preserves the nationally representative nature of the cohort and provides population-level estimates of disparities while ensuring visibility of each sexual identity subgroup.

*Abstract (page 2, line 34): “Compared to heterosexual participants, those identifying as gay or lesbian were more likely to be unemployed (prevalence ratio [PR] 2.05, 95% CI 1.01–4.14) and less likely to work in trades or manual occupations (PR 0.55, 95% CI 0.36–0.85) or in manufacturing and construction (PR 0.40, 95% CI 0.23–0.72). Bisexual participants had higher prevalence of labour force non-participation (PR 1.91, 95% CI 1.49–2.47) and unemployment (PR 2.05, 95% CI 1.24–3.38), and were less likely to work in agriculture, forestry or mining (PR 0.24, 95% CI 0.08–0.79). Participants of other sexual identities also had higher unemployment (PR 2.78, 95% CI 1.41–5.45). Longitudinally, bisexual participants were more likely to transition out of employment (incidence rate ratio [IRR] 2.08, 95% CI 1.35–3.21) and initiate paid sick leave (IRR 1.42, 95% CI 1.17–1.71), while gay or lesbian participants were more likely to commence working from home (IRR 1.72, 95% CI 1.21–2.44).”*

*Methods (page 8, line 184): “For the sexual identity analysis, each sexual identity group (gay or lesbian, bisexual, other) was compared to those identifying as heterosexual.”*

*Results – cross-sectional (page 10, line 229): “Compared to heterosexual participants, those identifying as gay or lesbian were younger, had higher education levels, and were more likely to be male, reside in major cities, and reside in areas of lower socioeconomic disadvantage (Table 1). Those identifying as bisexual were more likely to be younger and female compared to heterosexual participants, while those identifying with other sexual identities were more likely to reside in areas of higher socioeconomic disadvantage.*

*After adjusting for age, sex, highest education level, remoteness, and SEIFA, gay or lesbian participants were more likely to be unemployed (PR 2.05, 95% CI 1.01 – 4.14) and to work from home (PR 1.30, 95% CI 1.07 – 1.57) compared to heterosexual participants (Table 2). They were less likely to work in trades or manual occupations (PR 0.55, 95% CI 0.36 – 0.85) and to work in the secondary (manufacturing, electricity, gas, water and waste services, construction) sector (PR 0.40, 95% CI 0.23 – 0.72). Numerous differences in work participation outcomes were also identified between bisexual and heterosexual participants, after adjusting for age and sex: those identifying as bisexual had higher prevalence of labour force non-participation (PR 1.91, 95% CI 1.49 – 2.47), unemployment (PR 2.05, 95% CI 1.24 – 3.38), part-time employment (PR 1.22, 95% CI 1.03 – 1.45), and unpaid leave (PR 1.33, 95% CI 1.01 – 1.75). They were far less likely to work in the primary (agriculture, forestry, fishing, mining) sector (PR 0.24, 95% CI 0.08 – 0.79). After adjustment for SEIFA, those with other sexual identities were more likely to be unemployed (PR 2.78, 95% CI 1.41 – 5.45), part-time employed (PR 1.57, 95% CI 1.16 – 2.12), work in the public sector (PR 1.15, 95% CI 1.06 – 1.24), and work non-standard schedules (PR 1.60, 95% CI 1.36 – 1.88) compared to those identifying as heterosexual.”*

*Results – longitudinal (page 13, line 277): “As in the cross-sectional analysis, gay or lesbian participants were younger, had higher education levels, and were more likely to be male, reside in major cities, and reside in areas of lower socioeconomic disadvantage (S1 Table). Those identifying as bisexual were on average younger and more likely to be female compared to their heterosexual counterparts, while those of other sexual identity more commonly resided in areas of higher socioeconomic disadvantage. After adjusting for age, sex, highest education level, remoteness, and SEIFA, gay or lesbian participants were more likely to commence working from home during follow-up (IRR 1.72, 95% CI 1.21 – 2.44) compared to heterosexual participants (Table 3). Those identifying as bisexual made more frequent transitions out of employment (IRR 2.08, 95% CI 1.35 – 3.21) and made new use of paid sick leave (IRR 1.42, 95% CI 1.17 – 1.71) compared to those identifying as heterosexual, after adjusting for age and sex. No differences in work participation outcomes between those of other sexual identities and heterosexual participants were identified in the longitudinal analysis.”*

3. Regarding the Results and Discussion sections, I think the authors should first reconsider the consequences of comparing “otherness” or “non-heterosexual” and heterosexual participants before stating any outcomes that may be biased on the sample size used.

RESPONSE

As noted above, the Results section has been updated with comparisons of each sexual identity subgroup to those identifying as heterosexual. We have also revised the Discussion to comment on these findings among specific sexual minorities. Examples of amended text from the Discussion are presented below.

*Page 17, line 335: “Compared to heterosexual participants, those identifying as gay or lesbian were more likely to be unemployed and to work from home, and less likely to work in trades, manual occupations, or the secondary sector (manufacturing, construction, utilities). Bisexual participants were more likely to be outside the labour force, unemployed, and engaged in part-time or unpaid leave arrangements, and were under-represented in the primary industries of agriculture, forestry, fishing, and mining. Participants identifying with other sexual identities were more likely to be unemployed, work part-time, work in the public sector, and have non-standard work schedules. In longitudinal analyses, gay and lesbian participants were more likely than their heterosexual peers to commence working from home between 2020 and 2023, while bisexual participants had higher rates of transition out of employment and new use of paid sick leave.”*

*Page 18, line 363: “Notably, gay or lesbian participants in our study were less likely than heterosexual peers to leave school before completing high school, indicating greater persistence in pursuing secondary education. This suggests a possible strategic choice among gay or lesbian individuals to attain at least high school education in order to access occupations perceived as less stigmatised or more inclusive than diploma- or trade-based pathways, which often lead to heteronormative workplace cultures.”*

*Page 19, line 380: “Despite higher educational attainment, gay or lesbian participants had poorer work participation outcomes in both cross-sectional and longitudinal analyses. This discrepancy suggests the presence of systemic and structural barriers beyond individual qualifications, which aligns with previous evidence showing the negative impacts of workplace discrimination, exclusion, and harassment on LGBTQ+ employees’ career trajectories (7). At the personal level, these outcomes may relate to the mental and physical health impacts of minority stress (8). Numerous work participation disparities were also observed for bisexual participants. The term “double discrimination” has been used to describe the prejudice against bisexuals from both heterosexuals and the LGBTQ+ community (9). Community studies of bisexuals have shown higher rates of depression, anxiety, suicidality, and substance abuse, compared to monosexuals (10, 11). However, research on the specific workplace experiences of bisexuals is limited. For example, one small US survey found that over 50% of those identifying as bisexual reported employment discrimination at some point in their lives (12), which may in part explain the adverse work participation outcomes observed in our study. The increased likelihood of transitioning out of employment among bisexual individuals might reflect burnout or difficulties sustaining long-term career progression in unsupportive environments (13), as well as cumulative disadvantage at the societal level (14). Such cumulative disadvantage may manifest not only as stress-related health conditions but also as career attrition, whereby exposure to repeated marginalisation prompts withdrawal from certain roles, sectors, or leadership pathways.”*

*Page 21, line 437: “Although our sample of those with “other” sexual identities was also relatively small, we found that they had reduced labour force participation, higher unemployment, and higher likelihood of part-time employment. These findings underline the importance of co-designing research with people who hold these identities, as existing survey frameworks may inadequately capture the diversity of sexual identities such as asexual, pansexual, queer, or questioning. Without inclusive and community-informed measures, important differences in workplace participation and experiences may remain obscured, limiting the evidence base needed to inform equitable employment policy.”*

**Reviewer 3:**

1. While I do not have the statistical analysis skills to evaluate this manuscript from that perspective, I can say with confidence that the subject is logically presented, the rationales given for the verbal conclusions and recommendations appear well-reasoned, and the references and supporting articles cited represent a comprehensive sample of the relevant literature. This reviewer finds the article informative and insightful and supports its publication.

RESPONSE

**We appreciate your positive feedback and are grateful for your support of our manuscript’s publication.**

1.	Martino RJ, Krause KD, Griffin M, LoSchiavo C, Comer-Carruthers C, Halkitis PN. Employment Loss as a Result of COVID-19: a Nationwide Survey at the Onset of COVID-19 in US LGBTQ+ Populations. Sexuality Research and Social Policy. 2022;19(4):1855-66.

2.	Australian Bureau of Statistics. Estimates and characteristics of LGBTI+ populations in Australia. 2024. Accessed October 6, 2025. <https://www.abs.gov.au/statistics/people/people-and-communities/estimates-and-characteristics-lgbti-populations-australia/latest-release>.

3.	Gnambs T, Kaspar K. Disclosure of sensitive behaviors across self-administered survey modes: a meta-analysis. Behav Res Methods. 2015;47(4):1237-59.

4.	Tomic D, O’Dwyer M, Keegel T, Walker-Bone K. Mental health of LGBTQ+ workers: a systematic review. BMC Psychiatry. 2025;25(1):114.

5.	Oliveira A, Pereira H, Alckmin-Carvalho F. Occupational Health, Psychosocial Risks and Prevention Factors in Lesbian, Gay, Bisexual, Trans, Queer, Intersex, Asexual, and Other Populations: A Narrative Review. Societies. 2024;14(8).

6.	Maji S, Yadav N, Gupta P. LGBTQ+ in workplace: a systematic review and reconsideration. Equality, Diversity and Inclusion: An International Journal. 2024;43(2):313-60.

7.	Sears B, Castleberry NM, Lin A, Mallory C. LGBTQ People’s Experiences of Workplace Discrimination and Harassment. Williams Institute, School of Law, UCLA. 2024. Accessed June 18, 2025. <https://williamsinstitute.law.ucla.edu/publications/lgbt-workplace-discrimination/>.

8.	Hoy-Ellis CP. Minority Stress and Mental Health: A Review of the Literature. J Homosex. 2023;70(5):806-30.

9.	Arena DF, Jones KP. To “B” or not to “B”: Assessing the disclosure dilemma of bisexual individuals at work. J Vocat Behav. 2017;103:86-98.

10.	Ross LE, Salway T, Tarasoff LA, MacKay JM, Hawkins BW, Fehr CP. Prevalence of Depression and Anxiety Among Bisexual People Compared to Gay, Lesbian, and Heterosexual Individuals:A Systematic Review and Meta-Analysis. J Sex Res. 2018;55(4-5):435-56.

11.	Mongelli F, Perrone D, Balducci J, Sacchetti A, Ferrari S, Mattei G, et al. Minority stress and mental health among LGBT populations: An update on the evidence. Edizioni Minerva Medica. 2019;60:27-50.

12.	Tweedy A, Yescavage K. Employment Discrimination against Bisexuals and Others with Fluid Identities. SSRN Electronic Journal. 2013.

13.	Zindel Z, de Vries L. Career sacrifice for an LGBTQ*-friendly work environment? a choice experiment to investigate the job preferences of LGBTQ* people. PLoS One. 2024;19(6):e0296419.

14.	Matsick JL, Sullivan JT, Todd E, Kruk M, Cook JE. A social ecological approach to belonging in LGBTQ+ people. Nature Reviews Psychology. 2024;3(3):181-97.
