# Supplementary material for: Work participation disparities among LGBTQ+ Australians: Insights from a nationally representative cohort study
Source: PLoS One. 2026 Jan 14;21(1):e0339160. doi: 10.1371/journal.pone.0339160 (PMC12803448; doi:10.1371/journal.pone.0339160)
Supplement: Peer review history

# Peer review: Decision Letter 0

**DOI:** 10.1371/journal.pone.0339160.r001

**Authors:** Ozan Karadeniz

6 Oct 2025

Dear Dr. Tomic,

Thank you for submitting your manuscript to PLOS ONE. After careful consideration, we feel that it has merit but does not fully meet PLOS ONE’s publication criteria as it currently stands. Therefore, we invite you to submit a revised version of the manuscript that addresses the points raised during the review process.

Several major revisions are required before the manuscript can be considered for acceptance.Specifically, the authors must address [insert key methodological, reporting, or analytical issues raised by reviewers], which are essential to meet PLOS ONE’s publication criteria for methodological rigor and sufficient reporting transparency. These changes are required for acceptance.

In addition, there are several points raised by the reviewers regarding [e.g., clarity of writing, organization of figures/tables, contextualization of prior literature]. While these are not mandatory for acceptance, I strongly recommend that the authors address them to improve the readability and utility of the manuscript for future readers.

Where reviewer feedback differs, I advise the authors to prioritize the requests that ensure methodological validity, data transparency, and adherence to reporting standards, as these are aligned with PLOS ONE’s criteria. Suggestions that pertain primarily to style or depth of discussion should be considered optional but would strengthen the overall quality of the article.

Please submit your revised manuscript by  Nov 15 2025 11:59PM. If you will need more time than this to complete your revisions, please reply to this message or contact the journal office at plosone@plos.org . When you're ready to submit your revision, log on to https://www.editorialmanager.com/pone/ and select the 'Submissions Needing Revision' folder to locate your manuscript file.

- A rebuttal letter that responds to each point raised by the academic editor and reviewer(s). You should upload this letter as a separate file labeled 'Response to Reviewers'.
- A marked-up copy of your manuscript that highlights changes made to the original version. You should upload this as a separate file labeled 'Revised Manuscript with Track Changes'.
- An unmarked version of your revised paper without tracked changes. You should upload this as a separate file labeled 'Manuscript'.

If you would like to make changes to your financial disclosure, please include your updated statement in your cover letter. Guidelines for resubmitting your figure files are available below the reviewer comments at the end of this letter.

If applicable, we recommend that you deposit your laboratory protocols in protocols.io to enhance the reproducibility of your results. Protocols.io assigns your protocol its own identifier (DOI) so that it can be cited independently in the future. For instructions see: https://journals.plos.org/plosone/s/submission-guidelines#loc-laboratory-protocols . Additionally, PLOS ONE offers an option for publishing peer-reviewed Lab Protocol articles, which describe protocols hosted on protocols.io. Read more information on sharing protocols at https://plos.org/protocols?utm_medium=editorial-email&utm_source=authorletters&utm_campaign=protocols .

We look forward to receiving your revised manuscript.

Kind regards,

Ozan Karadeniz

Academic Editor

PLOS ONE

Journal Requirements:

When submitting your revision, we need you to address these additional requirements.

1. Please ensure that your manuscript meets PLOS ONE's style requirements, including those for file naming. The PLOS ONE style templates can be found at

https://journals.plos.org/plosone/s/file?id=wjVg/PLOSOne_formatting_sample_main_body.pdf and

https://journals.plos.org/plosone/s/file?id=ba62/PLOSOne_formatting_sample_title_authors_affiliations.pdf

2. We note that you have indicated that there are restrictions to data sharing for this study. PLOS only allows data to be available upon request if there are legal or ethical restrictions on sharing data publicly. For more information on unacceptable data access restrictions, please see http://journals.plos.org/plosone/s/data-availability#loc-unacceptable-data-access-restrictions.

Before we proceed with your manuscript, please address the following prompts:

a) If there are ethical or legal restrictions on sharing a de-identified data set, please explain them in detail (e.g., data contain potentially identifying or sensitive patient information, data are owned by a third-party organization, etc.) and who has imposed them (e.g., a Research Ethics Committee or Institutional Review Board, etc.). Please also provide contact information for a data access committee, ethics committee, or other institutional body to which data requests may be sent.

b) If there are no restrictions, please upload the minimal anonymized data set necessary to replicate your study findings to a stable, public repository and provide us with the relevant URLs, DOIs, or accession numbers. For a list of recommended repositories, please see

https://journals.plos.org/plosone/s/recommended-repositories. You also have the option of uploading the data as Supporting Information files, but we would recommend depositing data directly to a data repository if possible.

We will update your Data Availability statement on your behalf to reflect the information you provide.

3. If the reviewer comments include a recommendation to cite specific previously published works, please review and evaluate these publications to determine whether they are relevant and should be cited. There is no requirement to cite these works unless the editor has indicated otherwise. 

Additional Editor Comments:

The statistical methodology is sound and appropriate for the research questions. The main limitation is under-adjustment for confounders and limited power in the gender identity subgroup. Results should be interpreted with caution regarding causality and subgroup generalizability.

Strengths

• Correct statistical models (Poisson with robust SEs) → avoids overestimation of effects.

• Survey weighting accounted for representativeness.

• Both cross-sectional and longitudinal perspectives strengthen causal interpretation.

• Results clearly reported with confidence intervals.

⸻

Limitations / Points to Flag

1. Limited confounder adjustment – only age and education were included; other structural factors could influence results.

2. Small TGD sample size – increases risk of Type II error; underpowered for some outcomes.

3. Assumption of stable sexual identity over 3 years may not reflect reality.

4. Job satisfaction dichotomization loses nuance; ordinal or linear models could provide more detail.

5. Potential multiple testing issue – many outcomes tested, but no adjustment for multiple comparisons (risk of false positives).

[Note: HTML markup is below. Please do not edit.]

Reviewers' comments:

Reviewer's Responses to Questions

**Comments to the Author**

1. Is the manuscript technically sound, and do the data support the conclusions?

Reviewer #1: Yes

Reviewer #2: No

Reviewer #3: Yes

**********

2. Has the statistical analysis been performed appropriately and rigorously?

Reviewer #1: Yes

Reviewer #2: N/A

Reviewer #3: I Don't Know

**********

3. Have the authors made all data underlying the findings in their manuscript fully available?

The PLOS Data policy

Reviewer #1: Yes

Reviewer #2: Yes

Reviewer #3: Yes

**********

4. Is the manuscript presented in an intelligible fashion and written in standard English?

Reviewer #1: Yes

Reviewer #2: Yes

Reviewer #3: Yes

**********

Reviewer #1: Thank you for the opportunity to review the manuscript, “Work participation disparities among LGBTQ+ Australians: insights from a nationally representative cohort study.” The manuscript addresses an under-researched topic for LGBTQ+ individuals and warrants continued study.

Overall, the manuscript was well written and structured. The findings were clear, interesting, and provides a foundation for additional research. The discussion conceptualized the findings through established research-informed understandings of the community. This reviewers had minimal recommendations and questions.

- Page 7, Line 144: Recommend including an example or two of responses that were “inadequately described.”

- Page 9: I appreciated the explanation for why adjustments were not made for gender identity in the sexual identity analysis and similarly for sexual identity in the gender identity analysis. The rationale was clear and proactively addressed a question readers may have (i.e., what about individuals who identify as both non-heterosexual and TGD).

- Page 10, Lines 221-224 and Page 14, 279-280: How does the study’s sample sizes (% of non-heterosexual and TGD) compare to national population estimates? If possible, this reviewer recommends including that detail.

- Page 17, Line 329: The authors briefly reference self-disclosure hesitancy in surveys. This reviewer recommends expanding this as a potential limitation of the study because the findings do not capture those who chose not to self-disclose. It also may be worthwhile to include commentary about reasons for hesitancy to self-disclose on surveys.

Reviewer #2: Thank you for the opportunity to review the article “Work participation disparities among LGBTQ+ Australians: insights from a nationally representative cohort study”. This novel article examines work participation disparities among lesbian, gay, bisexual, transgender, queer, and other sexually and/or gender diverse (LGBTQ+) adults in Australia.

Here are some doubts/suggestions for consideration/reflection:

In line 77, instead of “facing”, I think the authors mean “regarding”.

Regarding the categorization of the sexually diverse participants, I find it contradictory and rather invisibilizing that in the introduction, the authors point out the fact that previous studies, and I quote them literally: “group LGBTQ+ workers together rather than disaggregating by sexual or gender identity, making it difficult to draw firm conclusions about the experiences of specific subgroups” and when they proceed to the analysis section, they go and do the same thing. I think that if the authors are writing a manuscript titled “Work participation disparities among LGBTQ+ Australians: insights from a nationally representative cohort study”, it doesn’t make sense that 94% of the sample they use is composed of heterosexual participants. I understand that LGBTQ+ people compose about 4.5% of the population in Australia, and obviously, heterosexual people are going to be the majority. However, as a researcher who also works in the field of sexual diversity, I believe that this article is poorly conceived. I think it would make much more sense to focus on the number of “non-heterosexual” participants (which I see is 929 people), divide them so that each category (bisexuals, lesbians, gays) can have the visibility it deserves, and take an equivalent sample of heterosexual people for comparison. In this way, the article would take on a more positive connotation than the ‘otherness’ it currently appears to have. I think the authors should consider this and modify it.

Regarding the Results and Discussion sections, I think the authors should first reconsider the consequences of comparing “otherness” or “non-heterosexual” and heterosexual participants before stating any outcomes that may be biased on the sample size used.

Overall, I think this manuscript is well written and could be useful. The authors should address the reviews, and then the manuscript will be greatly improved.

Reviewer #3: While I do not have the statistical analysis skills to evaluate this manuscript from that perspective, I can say with confidence that the subject is logically presented, the rationales given for the verbal conclusions and recomendations appear well-reasoned, and the references and supporting articles cited represent a comprehensive sample of the relevant literature. This reviewer finds the article informative and insightful and supports its publication.

**********

what does this mean? ). If published, this will include your full peer review and any attached files.

If you choose “no”, your identity will remain anonymous but your review may still be made public.

**Do you want your identity to be public for this peer review?** For information about this choice, including consent withdrawal, please see our Privacy Policy

Reviewer #1: No

Reviewer #2: No

Reviewer #3: No

**********

[NOTE: If reviewer comments were submitted as an attachment file, they will be attached to this email and accessible via the submission site. Please log into your account, locate the manuscript record, and check for the action link "View Attachments". If this link does not appear, there are no attachment files.]

While revising your submission, please upload your figure files to the Preflight Analysis and Conversion Engine (PACE) digital diagnostic tool, https://pacev2.apexcovantage.com/ . PACE helps ensure that figures meet PLOS requirements. To use PACE, you must first register as a user. Registration is free. Then, login and navigate to the UPLOAD tab, where you will find detailed instructions on how to use the tool. If you encounter any issues or have any questions when using PACE, please email PLOS at figures@plos.org . Please note that Supporting Information files do not need this step.

# Author comment: Author response to Decision Letter 1

**DOI:** 10.1371/journal.pone.0339160.r002

28 Oct 2025

27 Oct 2025

Dr Ozan Karadeniz

Academic Editor

PLOS ONE

Dear Dr Karadeniz,

Thank you for providing this helpful feedback, and for the opportunity to revise our manuscript. We have made the requested changes and address each of the editorial and reviewers’ comments below.

Associate Editor:

1. The statistical methodology is sound and appropriate for the research questions. The main limitation is under-adjustment for confounders and limited power in the gender identity subgroup. Results should be interpreted with caution regarding causality and subgroup generalizability. Limitations / Points to Flag:

• Limited confounder adjustment – only age and education were included; other structural factors could influence results.

• Small TGD sample size – increases risk of Type II error; underpowered for some outcomes.

• Assumption of stable sexual identity over 3 years may not reflect reality.

• Job satisfaction dichotomization loses nuance; ordinal or linear models could provide more detail.

• Potential multiple testing issue – many outcomes tested, but no adjustment for multiple comparisons (risk of false positives).

RESPONSE

Thank you for these constructive suggestions. We have revised the Limitations section to more clearly emphasise the need for cautious interpretation and to elaborate on the issues raised, including statistical power, potential Type II error, and multiple testing. In response to Reviewer 2’s comments, all sexual identity analyses were re-run with disaggregated sexual identity categories and additional covariate adjustment for age, sex, highest education level, remoteness, and area-level socioeconomic position (SEIFA), based on variables showing significant between-group differences. Adjusting only for covariates that differed meaningfully between groups helps to prevent statistical over-adjustment, which can remove variance intrinsic to sexual identity and obscure genuine disparities.

Page 22, line 451: “The small TGD sample size reduces statistical power in our analysis, increasing the likelihood of Type II error; findings for this group should therefore be interpreted cautiously. Sexual identity was assumed to remain stable between 2020 and 2023, which may not reflect the fluidity of sexual identity over time, particularly for younger cohorts. Potential social desirability bias may lead to underreporting of non-cisgender and non-heterosexual identities, both within the self-reported and interviewer-collected data, affecting the accuracy of our findings. Furthermore, the reliance on binary employment outcomes (e.g., employed vs. unemployed) may not capture more nuanced underemployment or job mismatch experiences, which are particularly relevant to LGBTQ+ individuals working in flexible, self-employed or precarious roles (1). Similarly, dichotomising job satisfaction may have reduced variability in responses; however, this was necessary given the small number of responses at each level of job satisfaction among specific sexual identities. Finally, given the exploratory scope and number of outcomes examined, we did not adjust for multiple comparisons; as such, some findings may reflect chance associations and should be interpreted with appropriate caution.”

Reviewer 1:

1. Page 7, Line 144: Recommend including an example or two of responses that were “inadequately described.”

RESPONSE

In light of this suggestion, we consulted with the HILDA Survey data custodians regarding examples of responses that were “inadequately described”; they advised that, due to confidentiality requirements, it is not possible to provide specific examples. As such, we have not included examples in the manuscript.

2. Page 9: I appreciated the explanation for why adjustments were not made for gender identity in the sexual identity analysis and similarly for sexual identity in the gender identity analysis. The rationale was clear and proactively addressed a question readers may have (i.e., what about individuals who identify as both non-heterosexual and TGD).

RESPONSE

Thank you for this positive feedback.

3. Page 10, Lines 221-224 and Page 14, 279-280: How does the study’s sample sizes (% of non-heterosexual and TGD) compare to national population estimates? If possible, this reviewer recommends including that detail.

RESPONSE

We have now included a paragraph in the Discussion comparing LGBTQ+ population estimates in our study to those reported by the Australian Bureau of Statistics, noting broad alignment and possible reasons for differences.

Page 18, line 351: “The proportion of LGBTQ+ respondents in this study broadly aligns with national population estimates. According to the ABS, in 2022, 3.6% of Australians aged 16 years and over identified as lesbian, gay, bisexual, or another non-heterosexual orientation, and 0.9% as TGD (2). Our weighted cross-sectional estimates of 5.9% and 1.2%, respectively, are slightly higher, which may be driven by several factors. Notably, in the HILDA Survey, questions on sexual and gender identity are included in a self-administered questionnaire, which could encourage greater disclosure compared with face-to-face ABS interviews. Differences in weighting processes, years of data collection (2020 and 2023 in our study vs. 2022 in the ABS data), and the small size of TGD populations making their estimates more sensitive to sampling variability, may also in part account for these differences.”

4. Page 17, Line 329: The authors briefly reference self-disclosure hesitancy in surveys. This reviewer recommends expanding this as a potential limitation of the study because the findings do not capture those who chose not to self-disclose. It also may be worthwhile to include commentary about reasons for hesitancy to self-disclose on surveys.

RESPONSE

We agree that self-disclosure hesitancy represents an important limitation of survey-based research. We have expanded the discussion to include this point and to outline potential reasons for nondisclosure, such as privacy concerns, fear of stigma or discrimination, and perceived lack of safety in certain contexts.

Page 19, line 374: “Self-disclosure hesitancy remains an important limitation of survey-based research (3), as individuals who choose not to disclose may differ systematically from those who do – for instance, they may have greater concerns about privacy, fear stigma or discrimination, or feel unsafe identifying as LGBTQ+ in certain contexts. Consequently, these dynamics may contribute to underestimation of disparities within population-level datasets.”

Reviewer 2:

1. In line 77, instead of “facing”, I think the authors mean “regarding”.

RESPONSE

We have updated the wording to “experienced by” to clarify that these risks are those that LGBTQ+ workers encounter, rather than risks merely discussed in the literature.

Page 3, line 74: “Reviews of the occupational health literature have identified multiple risks experienced by LGBTQ+ workers, including poorer mental health outcomes, elevated exposure to psychosocial hazards, lack of recognition in workplace policies, and limited access to inclusive organisational cultures (4-6).”

2. Regarding the categorization of the sexually diverse participants, I find it contradictory and rather invisibilizing that in the introduction, the authors point out the fact that previous studies, and I quote them literally: “group LGBTQ+ workers together rather than disaggregating by sexual or gender identity, making it difficult to draw firm conclusions about the experiences of specific subgroups” and when they proceed to the analysis section, they go and do the same thing. I think that if the authors are writing a manuscript titled “Work participation disparities among LGBTQ+ Australians: insights from a nationally representative cohort study”, it doesn’t make sense that 94% of the sample they use is composed of heterosexual participants. I understand that LGBTQ+ people compose about 4.5% of the population in Australia, and obviously, heterosexual people are going to be the majority. However, as a researcher who also works in the field of sexual diversity, I believe that this article is poorly conceived. I think it would make much more sense to focus on the number of “non-heterosexual” participants (which I see is 929 people), divide them so that each category (bisexuals, lesbians, gays) can have the visibility it deserves, and take an equivalent sample of heterosexual people for comparison. In this way, the article would take on a more positive connotation than the ‘otherness’ it currently appears to have. I think the authors should consider this and modify it.

RESPONSE

Thank you for raising this important point. We agree that disaggregating sexual identity subgroups is essential to avoid obscuring differences within the LGBTQ+ population. In response, we have now redone all sexual identity analyses to present results separately for each subgroup (gay or lesbian, bisexual, and other sexual identities) compared with heterosexual participants. We have updated the relevant tables (Tables 1-3; S1 Table) and text accordingly. We believe this approach is preferable to creating a matched heterosexual sample, as it preserves the nationally representative nature of the cohort and provides population-level estimates of disparities while ensuring visibility of each sexual identity subgroup.

Abstract (page 2, line 34): “Compared to heterosexual participants, those identifying as gay or lesbian were more likely to be unemployed (prevalence ratio [PR] 2.05, 95% CI 1.01–4.14) and less likely to work in trades or manual occupations (PR 0.55, 95% CI 0.36–0.85) or in manufacturing and construction (PR 0.40, 95% CI 0.23–0.72). Bisexual participants had higher prevalence of labour force non-participation (PR 1.91, 95% CI 1.49–2.47) and unemployment (PR 2.05, 95% CI 1.24–3.38), and were less likely to work in agriculture, forestry or mining (PR 0.24, 95% CI 0.08–0.79). Participants of other sexual identities also had higher unemployment (PR 2.78, 95% CI 1.41–5.45). Longitudinally, bisexual participants were more likely to transition out of employment (incidence rate ratio [IRR] 2.08, 95% CI 1.35–3.21) and initiate paid sick leave (IRR 1.42, 95% CI 1.17–1.71), while gay or lesbian participants were more likely to commence working from home (IRR 1.72, 95% CI 1.21–2.44).”

Methods (page 8, line 184): “For the sexual identity analysis, each sexual identity group (gay or lesbian, bisexual, other) was compared to those identifying as heterosexual.”

Results – cross-sectional (page 10, line 229): “Compared to heterosexual participants, those identifying as gay or lesbian were younger, had higher education levels, and were more likely to be male, reside in major cities, and reside in areas of lower socioeconomic disadvantage (Table 1). Those identifying as bisexual were more likely to be younger and female compared to heterosexual participants, while those identifying with other sexual identities were more likely to reside in areas of higher socioeconomic disadvantage.

After adjusting for age, sex, highest education level, remoteness, and SEIFA, gay or lesbian participants were more likely to be unemployed (PR 2.05, 95% CI 1.01 – 4.14) and to work from home (PR 1.30, 95% CI 1.07 – 1.57) compared to heterosexual participants (Table 2). They were less likely to work in trades or manual occupations (PR 0.55, 95% CI 0.36 – 0.85) and to work in the secondary (manufacturing, electricity, gas, water and waste services, construction) sector (PR 0.40, 95% CI 0.23 – 0.72). Numerous differences in work participation outcomes were also identified between bisexual and heterosexual participants, after adjusting for age and sex: those identifying as bisexual had higher prevalence of labour force non-participation (PR 1.91, 95% CI 1.49 – 2.47), unemployment (PR 2.05, 95% CI 1.24 – 3.38), part-time employment (PR 1.22, 95% CI 1.03 – 1.45), and unpaid leave (PR 1.33, 95% CI 1.01 – 1.75). They were far less likely to work in the primary (agriculture, forestry, fishing, mining) sector (PR 0.24, 95% CI 0.08 – 0.79). After adjustment for SEIFA, those with other sexual identities were more likely to be unemployed (PR 2.78, 95% CI 1.41 – 5.45), part-time employed (PR 1.57, 95% CI 1.16 – 2.12), work in the public sector (PR 1.15, 95% CI 1.06 – 1.24), and work non-standard schedules (PR 1.60, 95% CI 1.36 – 1.88) compared to those identifying as heterosexual.”

Results – longitudinal (page 13, line 277): “As in the cross-sectional analysis, gay or lesbian participants were younger, had higher education levels, and were more likely to be male, reside in major cities, and reside in areas of lower socioeconomic disadvantage (S1 Table). Those identifying as bisexual were on average younger and more likely to be female compared to their heterosexual counterparts, while those of other sexual identity more commonly resided in areas of higher socioeconomic disadvantage. After adjusting for age, sex, highest education level, remoteness, and SEIFA, gay or lesbian participants were more likely to commence working from home during follow-up (IRR 1.72, 95% CI 1.21 – 2.44) compared to heterosexual participants (Table 3). Those identifying as bisexual made more frequent transitions out of employment (IRR 2.08, 95% CI 1.35 – 3.21) and made new use of paid sick leave (IRR 1.42, 95% CI 1.17 – 1.71) compared to those identifying as heterosexual, after adjusting for age and sex. No differences in work participation outcomes between those of other sexual identities and heterosexual participants were identified in the longitudinal analysis.”

3. Regarding the Results and Discussion sections, I think the authors should first reconsider the consequences of comparing “otherness” or “non-heterosexual” and heterosexual participants before stating any outcomes that may be biased on the sample size used.

RESPONSE

As noted above, the Results section has been updated with comparisons of each sexual identity subgroup to those identifying as heterosexual. We have also revised the Discussion to comment on these findings among specific sexual minorities. Examples of amended text from the Discussion are presented below.

Page 17, line 335: “Compared to heterosexual participants, those identifying as gay or lesbian were more likely to be unemployed and to work from home, and less likely to work in trades, manual occupations, or the secondary sector (manufacturing, construction, utilities). Bisexual participants were more likely to be outside the labour force, unemployed, and engaged in part-time or unpaid leave arrangements, and were under-represented in the primary industries of agriculture, forestry, fishing, and mining. Participants identifying with other sexual identities were more likely to be unemployed, work part-time, work in the public sector, and have non-standard work schedules. In longitudinal analyses, gay and lesbian participants were more likely than their heterosexual peers to commence working from home between 2020 and 2023, while bisexual participants had higher rates of transition out of employment and new use of paid sick leave.”

Page 18, line 363: “Notably, gay or lesbian participants in our study were less likely than heterosexual peers to leave school before completing high school, indicating greater persistence in pursuing secondary education. This suggests a possible strategic choice among gay or lesbian individuals to attain at least high school education in order to access occupations perceived as less stigmatised or more inclusive than diploma- or trade-based pathways, which often lead to heteronormative workplace cultures.”

Page 19, line 380: “Despite higher educational attainment, gay or lesbian participants had poorer work participation outcomes in both cross-sectional and longitudinal analyses. This discrepancy suggests the presence of systemic and structural barriers beyond individual qualifications, which aligns with previous evidence showing the negative impacts of workplace discrimination, exclusion, and harassment on LGBTQ+ employees’ career trajectories (7). At the personal level, these outcomes may relate to the mental and physical health impacts of minority stress (8). Numerous work participation dispari

Attachment Submitted filename: Reviewer responses 27.10.25.docx

# Peer review: Decision Letter 1

**DOI:** 10.1371/journal.pone.0339160.r003

**Authors:** Ozan Karadeniz

3 Dec 2025

Work participation disparities among LGBTQ+ Australians: insights from a nationally representative cohort study

PONE-D-25-34839R1

Dear Dr. Dunya Tomic,

We’re pleased to inform you that your manuscript has been judged scientifically suitable for publication and will be formally accepted for publication once it meets all outstanding technical requirements.

Within one week, you’ll receive an e-mail detailing the required amendments. When these have been addressed, you’ll receive a formal acceptance letter and your manuscript will be scheduled for publication.

An invoice will be generated when your article is formally accepted. Please note, if your institution has a publishing partnership with PLOS and your article meets the relevant criteria, all or part of your publication costs will be covered. Please make sure your user information is up-to-date by logging into Editorial Manager at Editorial Manager®  and clicking the ‘Update My Information' link at the top of the page. For questions related to billing, please contact billing support .

If your institution or institutions have a press office, please notify them about your upcoming paper to help maximize its impact. If they’ll be preparing press materials, please inform our press team as soon as possible -- no later than 48 hours after receiving the formal acceptance. Your manuscript will remain under strict press embargo until 2 pm Eastern Time on the date of publication. For more information, please contact onepress@plos.org.

Kind regards,

Ozan Karadeniz

Academic Editor

PLOS ONE

Additional Editor Comments (optional):

We are pleased to inform you that your manuscript titled "Work participation disparities among LGBTQ+ Australians: insights from a nationally representative cohort study" has been accepted for publication in Plos ONE.

Your submission demonstrated a significant contribution to understanding employment disparities within the LGBTQ+ community in Australia, and we commend the rigor and clarity of your analysis.

The manuscript will now proceed to the production stage, and our editorial team will contact you shortly regarding proofs, final formatting, and publication timelines.

We appreciate your contribution to advancing research in this important area and look forward to sharing your work with our readership.

Reviewers' comments:

Reviewer's Responses to Questions

**Comments to the Author**

Reviewer #1: All comments have been addressed

Reviewer #2: All comments have been addressed

Reviewer #3: All comments have been addressed

**********

2. Is the manuscript technically sound, and do the data support the conclusions?

Reviewer #1: Yes

Reviewer #2: Yes

Reviewer #3: Yes

**********

3. Has the statistical analysis been performed appropriately and rigorously?

Reviewer #1: I Don't Know

Reviewer #2: Yes

Reviewer #3: Yes

**********

4. Have the authors made all data underlying the findings in their manuscript fully available?

The PLOS Data policy

Reviewer #1: Yes

Reviewer #2: No

Reviewer #3: Yes

**********

5. Is the manuscript presented in an intelligible fashion and written in standard English?

Reviewer #1: Yes

Reviewer #2: Yes

Reviewer #3: Yes

**********

Reviewer #1: Thank you for the opportunity to review the revised manuscript, “Work participation disparities among LGBTQ+ Australians: insights from a nationally representative cohort study.” The authors were clear in how they addressed each reviewer’s feedback and noted location in the manuscript.

The incorporated feedback in the manuscript appropriately expanded the acknowledged limitations, provide additional context for findings and interpretations, reflects diversity of the identity categories, and has enhanced readability from rephrasing targeted sections. This reviewer supports publication and believes readers will find the manuscript informative.

Reviewer #2: Thank you for the opportunity to re-review this article. The authors have addressed correctly all comments made previously and I have no further comments.

Reviewer #3: This reviewer appreciates the Authors' detailed responses to all the reviewers' comments and their revisions to the original rext.

**********

what does this mean? ). If published, this will include your full peer review and any attached files.

If you choose “no”, your identity will remain anonymous but your review may still be made public.

**Do you want your identity to be public for this peer review?** For information about this choice, including consent withdrawal, please see our Privacy Policy

Reviewer #1: No

Reviewer #2: No

Reviewer #3: No

**********

# Editor report: Acceptance letter

**DOI:** 10.1371/journal.pone.0339160.r004

**Authors:** Ozan Karadeniz

PONE-D-25-34839R1

PLOS One

Dear Dr. Tomic,

I'm pleased to inform you that your manuscript has been deemed suitable for publication in PLOS One. Congratulations! Your manuscript is now being handed over to our production team.

At this stage, our production department will prepare your paper for publication. This includes ensuring the following:

* All references, tables, and figures are properly cited

* All relevant supporting information is included in the manuscript submission,

* There are no issues that prevent the paper from being properly typeset

You will receive further instructions from the production team, including instructions on how to review your proof when it is ready. Please keep in mind that we are working through a large volume of accepted articles, so please give us a few days to review your paper and let you know the next and final steps.

Lastly, if your institution or institutions have a press office, please let them know about your upcoming paper now to help maximize its impact. If they'll be preparing press materials, please inform our press team within the next 48 hours. Your manuscript will remain under strict press embargo until 2 pm Eastern Time on the date of publication. For more information, please contact onepress@plos.org.

You will receive an invoice from PLOS for your publication fee after your manuscript has reached the completed accept phase. If you receive an email requesting payment before acceptance or for any other service, this may be a phishing scheme. Learn how to identify phishing emails and protect your accounts at https://explore.plos.org/phishing.

If we can help with anything else, please email us at customercare@plos.org.

Thank you for submitting your work to PLOS ONE and supporting open access.

Kind regards,

PLOS ONE Editorial Office Staff

on behalf of

MD Ozan Karadeniz

Academic Editor

PLOS One
